# Echocardiographic evaluation of mitral geometry in functional mitral regurgitation

**DOI:** 10.1186/1749-8090-3-54

**Published:** 2008-10-07

**Authors:** Anita Sadeghpour, Firoozeh Abtahi, Majid Kiavar, Maryam Esmaeilzadeh, Niloofar Samiei, Seyedeh Zahra Ojaghi, Hooman Bakhshandeh, Majid Maleki, Feridoun Noohi, Ahmad Mohebbi

**Affiliations:** 1 Department of Cardiovascular Medicine, Echocardiography Lab. Rajaei Cardiovascular Medical and Research Center, adjacent to Mellat Park, Tehran, Iran; 2 Department of Cardiovascular Medicine, Rajaei Cardiovascular Medical and Research Center, adjacent to Mellat Park, Tehran, Iran

## Abstract

**Objectives:**

We sought to evaluate the geometric changes of the mitral leaflets, local and global LV remodeling in patients with left ventricular dysfunction and varying degrees of Functional mitral regurgitation (FMR).

**Background:**

Functional mitral regurgitation (FMR) occurs as a consequence of systolic left ventricular (LV) dysfunction caused by ischemic or nonischemic cardiomyopathy. Mitral valve repair in ischemic MR is one of the most controversial topic in surgery and proper repairing requires an understanding of its mechanisms, as the exact mechanism of FMR are not well defined.

**Methods:**

136 consecutive patients mean age of 55 with systolic LV dysfunction and FMR underwent complete echocardiography and after assessing MR severity, LV volumes, Ejection Fraction, LV sphericity index, C-Septal distance, Mitral valve annulus, Interpapillary distance, Tenting distance and Tenting area were obtained.

**Results:**

There was significant association between MR severity and echocardiogarphic indices (all p values < 0.001). Severe MR occurred more frequently in dilated cardiomyopathy (DCM) patients compared to ischemic patients, (p < 0.001). Based on the model, only Mitral valve tenting distance (TnD) (OR = 22.11, CI 95%: 14.18 – 36.86, p < 0.001) and Interpapillary muscle distance (IPMD), (OR = 6.53, CI 95%: 2.10 – 10.23, p = 0.001) had significant associations with MR severity.

Mitral annular dimensions and area, C-septal distance and sphericity index, although greater in patients with severe regurgitation, did not significantly contribute to FMR severity.

**Conclusion:**

Degree of LV enlargement and dysfunction were not primary determinants of FMR severity, therefore local LV remodeling and mitral valve apparatus deformation are the strongest predictors of functional MR severity.

## Introduction

Functional Mitral Regurgitation (FMR) defined as a regurgitation that seen with structurally normal valve leaflets. It occurs as a consequence of systolic LV dysfunction caused by ischemic or nonischemic cardiomyopathy. Functional MR occurs in roughly 20–25% of patients following MI and 50% of those with CHF [1,2]. Any degree of FMR in patients with LV dysfunction conveys adverse prognosis, [2-8], with a graded relationship between severity of regurgitation and reduced survival [2,4]. Although global LV dilation and sphericity has been attributed to FMR by alteration in mitral annulus and subvalvular apparatus, and ultimately incomplete coaptation, but local LV remodeling and mitral valve deformation seems to be more important determinants of FMR severity. It is also suggested from propensity for recurrence of FMR following ring annuluplasty, [1-3,6-11]and addressed adjunctive surgical operative techniques to correct subvalvular changes as a major target to prevent residual or recurrent regurgitation following mitral valve repair procedures [1,3,11,12]. Optimal repairing of FMR requires an understanding of its mechanisms and it may be useful to use some echocardiographic predictors as a surrogate for its potential severity and its major mechanism for planning therapeutic options. Therefore, we undertook a quantitative study of patients with LV dysfunction in order to determine the most important determinants of FMR severity.

## Methods

### Study population

This study was conducted in Rajaei cardiovascular medical and research center, Tehran, Iran, where is one of the major referral center of cardiovascular diseases in the country.136 patients (mean age (SD) 55 (13.5) years, 85 (63%) male) with systolic LV dysfunction (mean EF (SD) 20% (8.7%)) and functional MR were enrolled. This study was approved by the ethics committee of Rajaei cardiovascular medical and research center. Inclusion criteria were: (1) systolic LV dysfunction (LVEF less than 50%); (2) structurally normal cardiac valves; (3)adequate 2D image for quantification of LV remodeling and mitral apparatus deformation; and (4)sinus rhythm. Exclusion criteria were (1) clinical or echocardiographic evidence of other cardiac diseases, such as organic valvular, pericardial, congenital, or infiltrative heart disease; (2) more than trace aortic regurgitation; (3) acute myocardial infarction; (4) suboptimal echocardiographic windows, leading to incomplete quantification of FMR or anatomic assessment; and (5) atrial fibrillation or flutter.

### Echocardiographic measurements

All patients underwent complete transthoracic echocardiography for assessing MR severity and measuring the indices of global and local LV remodeling as well as mitral deformation indexes. All exams performed using Vivid seven (GE Vingmed ultrasound), with a 3S probe.

### Evaluation of Mitral Regurgitation

**Figure 1:**
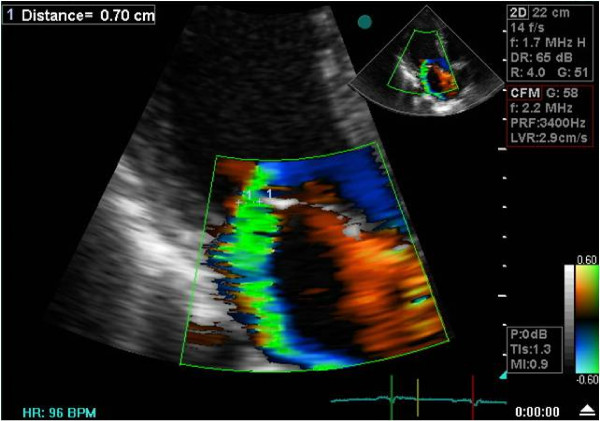
**Echocardiographic assessment of MR severity by VC**. Color flow imaging of mitral regurgitation for measurement of vena contracta in apical long axis view.

**Figure 2:**
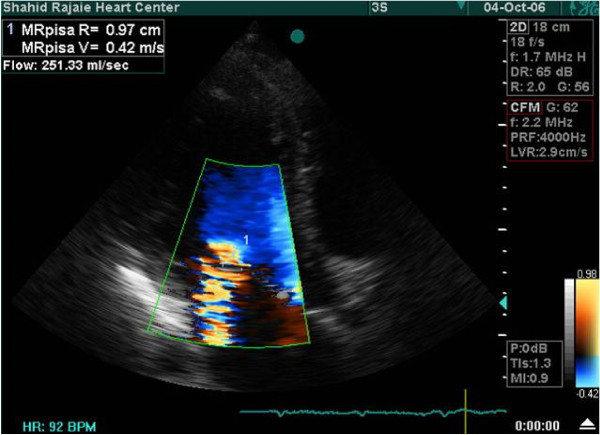
**Echocardiographic assessment of MR severity by PISA**. Measurement of PISA radius, ERO and regurgitant volume for assessment of MR severity using color Doppler imaging and CW Doppler.

Complete 2D and color Doppler flow imaging were performed according to guidelines of the ASE, to determine severity of the mitral regurgitation ^14^.ERO and MR volume assessed by PISA and VC methods (Figure 1, 2).

**Figure 3:**
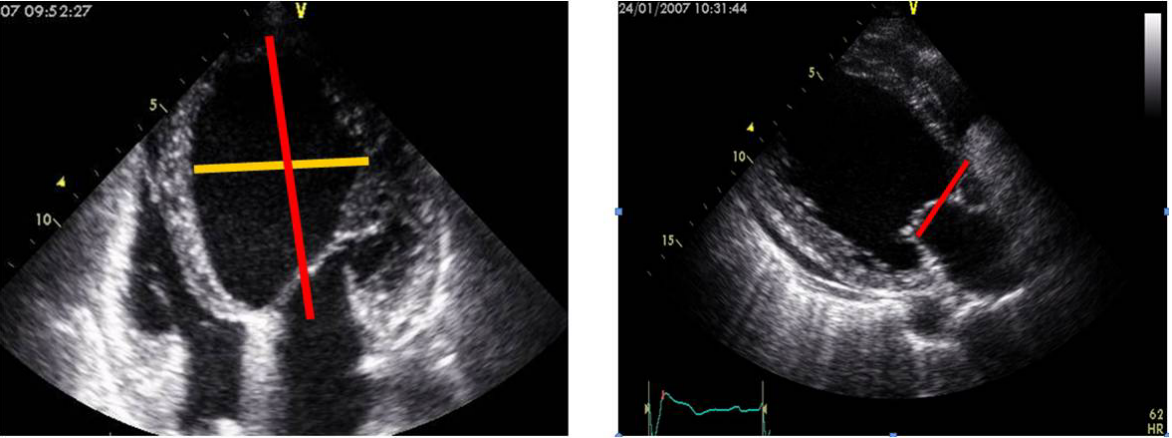
**Measurement of LV sphericity index**. LV sphericity index in apical 4-chamber view (3a), and C-septal distance in parasternal long-axis view (3b).

ERO ≥ 0.2 cm^2 ^defined as severe MR(significant FMR). In patients with lesser degree of MR (less than mild to moderate) assessment of PISA wasnot done due to lesser accuracy in these patient. Measurements of LV indices: LV dimensions was measured at diastole and systole (LVEDD, LVESD) in parasternal long axis view. LV volumes and EF were obtained by biplane Simpson's method. Patterns of regional wall motion abnormalities were assessed according to a 16 segment model [13]. LV sphericity was measured by the LV short-to-long-axis dimension ratio in end-diastolic apical four-chamber view (Figure 3a). LA volume was measured by tracing LA cavity at end-systolic four-chamber and two-chamber views, [LA Volume = (0.85 × A1 × A2)÷L].

C-Septal distance was measured at mid-systolic parasternal long-axis view; The distance was measured between septum at hinge point of aortic valve cusp and coaptation point of mitral valve leaflets (Figure 3b).

Mitral valve annulus was measured at mid-diastolic four-chamber and parasternal long-axis views. Mitral annular area was measured by formula; (MA area = d1 × d2 × π/4).

Interpapillary distance was measured at diastolic mid parasternal short-axis view as a distance between two papillary muscles tips.

**Figure 4:**
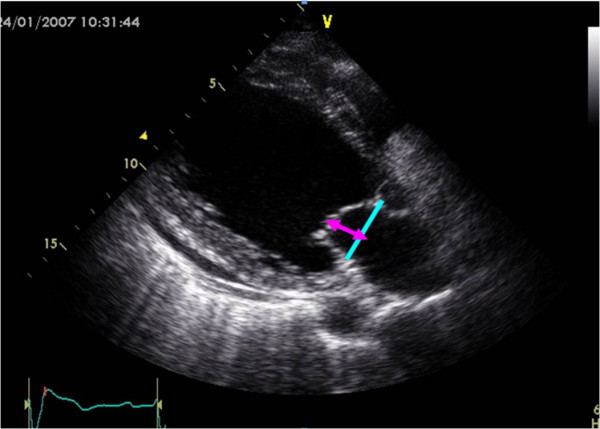
**Measurement of mitral deformation indexes**. Tenting distance in parasternal long-axis view.

Mitral Deformation Indexes: Tenting distance was measured as a distance between coaptation point of mitral leaflets and annular plane of MV at mid-systolic parasternal long axis view. Tenting area was measured as an area enclosed between leaflets and annular plane. (Figure 4).

### Statistical analysis

One-way ANOVA (and Bonferroni post-hoc test) and chi-square tests were used for comparison of data among the groups. P-value < 0.05 considered as statistically significant. An ordinal logistic regression (OLR) model was fitted to study the adjusted association between severity of FMR and other echocardiogarphic and background determinants. The variables selected based on theoretic considerations and their statistically significant associations in bivariate analysis. STATA™/SE 8 (STATA Corporation, Texas, USA) was used for data analysis.

## Results

### Baseline Characteristics

Of total 136 patients enrolled in this study, 85 (63%) were male. Mean (SD) age was 55(13.5) years. 51 patients (37.5%) presented with mild regurgitation, 39 patients (28.7%) with moderate and 46 patients (33.8%) with severe functional mitral regurgitation.104 (77%) patients categorized as ischemic cardiomyopathy (ICM) by documented history of infarction and/or coronary angiography and 32 (24%) of them as dilated cardiomyopathy (DCM). All of them had LV systolic dysfunction (mean (SD) EF = 23% (9%), range 10% to 50%). Mean (SD) of end-systolic diameter (ESD), end-systolic volume (ESV), end-diastolic diameter (EDD) and end-diastolic volume (EDV) of LV were: 5.50 (0.89) cm, 128.19 (53.22) ml, 6.49 (0.75) cm and 166.99 (57.71) ml, respectively. Mean volume (SD) of LA was 72.71 (26) ml. Also, the mean pressure (SD) of pulmonary artery (PAP) was 45 (15) mmHg.

Associations between these characteristics and severity of FMR are shown in table 1. Comparing disease category for FMR severity revealed that mild degree of regurgitation was more frequent in ICM, and in contrast severe regurgitation occurred more frequently in DCM (P = 0.02). Systolic function of LV became worse in more severe degrees of FMR (p < 0.001); however EF in patients with moderate and severe FMR had no significant difference (p = 0.95) and the observed significant difference belonged to the difference between the mean of EF in mild FMR and severe forms of FMR. Systolic and diastolic dimensions and volumes of LV had significant associations with progression of FMR severity. More severe degrees of FMR had greater means of dimensions/volumes (all p values less than 0.02). The same associations were shown between volume of LA, pressure of pulmonary artery and FMR severity. The only exception was LVESD, that no significant difference was seen between moderate and severe groups (p value = 0.13).

**Table 1:** Baseline characteristics

	MILD	MODERATE	SEVERE
Total (33.8%)	51(37.5%)	39 (28.7%)	46
Age 52.6 ± 15.2	55.6 ± 12.6	56.7 ± 13.2	
Sex (M/F) 26/20	37/14	22/17	
Disease			
ICM	47	28	29
DCM	4	11	17
Previous MI			
None	4	10	17
Anterior	31	16	15
Inferior	12	3	9
Both	3	3	2
Angiography			
SVD	7	2	2
2VD	12	4	5
3VD	11	7	8
Normal	3	7	10
Previous CABG	5	8	9

ICM: ischemic cardiomyopathy, DCM: Dilated cardiomyopathy, MI: Myocardial Infarction, SVD: single vessel disease, 2VD: two vessel disease, 3VD: three vessel disease, CABG: coronary artery bypass graft.

### LV Remodeling and MV Deformation Indices

**Figure 5:**
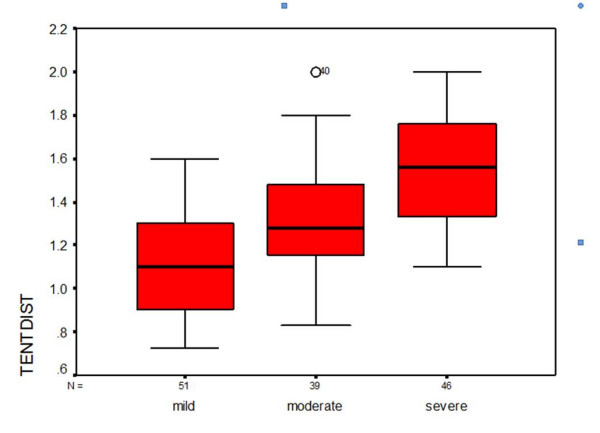
**Diagram of Tenting distance**. Correlation between Tenting distance as a measure of mitral deformation and FMR severity.

All echocardiogarphic measurements of LV remodeling and mitral valve deformation are shown in table 2. All of the indices had an association with severity of FMR. Mitral annulus dimension in two views, as well as mitral annular area were higher in presence of greater degree of regurgitation. Mitral deformation indices (tenting distance and tenting area) increased significantly with higher degree of FMR (P < 0.001), (Figure 5). Interpapillary muscle distance also increased significantly in patients with severe regurgitation (P < 0.001). Multivariate analysis was performed by fitting an ordinal logistic regression model to determine the adjusted associations between FMR severity (as dependent variable) and other variables, which are participating in the pathophysiology of LV remodeling and MV deformation. The results are presented in table 3. As it is shown, after adjustment, only tenting distance (OR = 14.01 [CI 95%: 2.39–82.09]; p = 0.003), interpapillary muscle distance (OR = 3.67 [CI 95%: 1.04–12.91]; p = 0.043) and left atrial volume (OR = 1.05 [CI 95%: 1.02–1.07]; p value < 0.001) had significant associations with FMR severity. According to point estimate OR, tenting distance had the most strong relationship among them, however, the wide confidence interval made the conclusion questionable.

**Table 2:** Echocardiographic measurements

PATIENTS CLASSIFICATION		P VALUE by ANOVA		
	MILD	MODERATE	SEVERE	P

EF (%)	27.9 ± 8.25	19.8 ± 6.85	20.5 ± 7.7	< 0.001
PAP (mmHg)	34.9 ± 13.4	46.7 ± 13	54.5 ± 11.2	< 0.001
LVEDD(cm)	6.07 ± 0.65	6.5 ± 0.73	6.9 ± 0.62	< 0.001
LVESD(cm)	4.9 ± 0.75	5.6 ± 0.9	5.98 ± 0.71	< 0.001
LVEDV(ml)	141 ± 47.7	165 ± 56.4	196 ± 56	< 0.001
LVESV(ml)	101 ± 41	131 ± 52	155 ± 52	< 0.001
Sph index	0.65 ± 0.09	0.71 ± 0.07	0.73 ± 0.09	< 0.001
LA vol(ml)	54.9 ± 19.4	71.9 ± 17.5	93.1 ± 23.6	< 0.001
MAD(PSL)cm	3.07 ± 0.33	3.35 ± 0.28	3.58 ± 0.45	< 0.001
MAD(4C)cm	3.13 ± 0.31	3.22 ± 0.27	3.43 ± 0.32	< 0.001
MAA(cm2)	7.6 ± 1.5	8.5 ± 1.25	9.7 ± 1.95	< 0.001
TnD(cm)	1.1 ± 0.25	1.32 ± 0.27	1.56 ± 0.25	< 0.001
Tn A(cm2)	1.68 ± 0.53	2.35 ± 0.77	2.81 ± 0.68	< 0.001
Inter PMD(cm)	2.37 ± 0.4	3 ± 0.52	3.23 ± 0.5	< 0.001
C-septal D(cm)	3.8 ± 0.47	4 ± 0.41	4.35 ± 0.41	< 0.001

EF: ejection fraction, PAP: pulmonary arterial pressure, Sph: sphericity, MAD: mitral annular diameter, MAA; mitral annular area, TnD: tenting distance, TnA: tenting area, PMD: papillary muscle distance.

**Table 3:** Analysis of echocardiographic parameters by ordinal logistic regression method.

Severity |	Coef.	Std. Err.	z	P > |z|	[95% Conf. Interval]	
LVEDD |	-.9521943	.974159	-0.98	0.328	-2.861511	.9571223
LVESD |	.5023955	.7797728	0.64	0.519	-1.025931	2.030722
LVEDV |	-.0088845	.0198006	-0.45	0.654	-.0476929	.0299239
LVESV |	.0101455	.0220899	0.46	0.646	-.0331499	.053441
Sph index |	1.694894	2.995477	0.57	0.572	-4.176134	7.565922
LA volume |	.050798	.0128922	3.94	0.000	.0255298	.0760662
MAD(PSL) |	-.1327646	3.45583	-0.04	0.969	-6.906067	6.640538
MAD(4C) |	-3.56701	3.376422	-1.06	0.291	10.18468	3.050656
MAA |	.5526684	1.323051	0.42	0.676	-2.040464	3.145801
TnD |	2.298142	0.895525	2.6	0.009	.5625475	4.03374
TnA |	.618084	.3694216	1.67	0.094	-.1059689	1.342137
Inter PMD |	1.332556	.5494736	2.40	0.014	.275075	2.389905
C-septal D |	-.1284093	.8348227	-0.15	0.878	-1.764632	1.507813
cut1 |	.1641684	11.30231	(Ancillary parameters)			
_cut2 |	2.442575	11.31522				

Sph: sphericity, MAD: mitral annular diameter, MAA; mitral annular area, TnD: tenting distance, TnA: tenting area, PMD: papillary muscle distance.

Based on OLR model, we estimated the values for tenting distance index, as an important predictor of FMR severity, in pre-defined probabilities of FMR. It can be seen that tenting distance 0.45 cm predicts mild FMR and 2.35 cm predicts severe FMR with 80% probability.

## Discussion

The present study shows that mitral deformation in patients with systolic dysfunction provides reliable suggestion of valves subject to functional regurgitation, with a graded relationship between valve deformation indices and FMR severity. In early experimental studies, FMR has been attributed to global LV dilatation or sphericity. Later, animal studies have suggested complex alterations of spatial relationship between left ventricle and mitral apparatus to induce functional MR [8,15]. In patients with LV dysfunction and dilation, the leaflets are usually tethered by outward displacement of the left ventricular wall and papillary muscles [4,8].

The papillary muscles which are normally parallel to the LV long axis, displaced as a result of ischemia or heart failure, drawing the leaflets into the ventricle and restricting their motion toward closure [2,8]. In the present study, tenting distance of mitral valve was the strongest independent determinant of functional mitral regurgitation severity as tenting distance 0.45 cm can predict 80% probability of being in mild FMR and tenting distance 2.35 cm can predict 80% probability of being in severe FMR. Several studies were demonstrated that tethering length is the final common pathway determining the amount of regurgitation [1-3,6-11,15-17]. In 128 patients with LV dysfunction, Yiu et al found that systolic mitral tenting area was strongly correlated with FMR severity and larger ERO was associated with greater amount of regurgitation [8]. By real-time 3 dimensional echocardiography geometric components of mitral valve deformation could be assessed more accurately [7,9,10,13,14]. Using 3D echocardiography, mitral valve tenting volume (TnV) as a clinical parameter of FMR, evaluated in 31 patients with LV systolic dysfunction by Song et al [18]. They found that minimal TnV was the only independent determinant of ERO, and value of minimal TnV ≥ 3.9 ml diagnosed significant FMR with high sensitivity and specificity[18].

We sought that interpapillary muscle distance contributed independently to functional MR severity. Other studies also showed interpapillary distance and displacement of papillary muscles as a reliable indicator of FMR, correlated with regurgitation severity [6,15-18]. Yiu et al showed that posterior displacement of PMs as a result of local LV remodeling contributed independently to larger ERO of FMR [8]. So anatomical position of papillary muscles should be an important factor in assessment of mechanism of functional MR. Also dynamic shift of papillary muscle position during aggressive treatment for heart failure was associated with reduction of valve tethering and regurgitation [9]. Significant FMR occurs more commonly in patients with posterior than anterior infarction [1,7,15]. In this study, There was no difference in MR severity between patients with the history of anterior and postero-inferior infarction, but comparing disease category for FMR severity mild degree of regurgitation was more frequently in ICM, and in contrast severe regurgitation occurred more frequently in DCM. Agricola et al demonstrated the effect of tethering pattern on significance of ischemic MR. They showed that pattern of asymmetric posterior tethering as a result of local LV remodeling was usually associated with inferior infarction and is the major determinant of systolic mitral valve tenting and functional MR [17]. In our study mitral annular area has been greater in patients with severe FMR, but it did not contributed independently to regurgitation severity. In a study by Popvic et al using 3D echocardiography in animal dilated cardiomyopathy model, annular dilation was the strongest predictor of functional MR and regurgitation volume [5]. However other studies showed that annular dilation as an associated lesion contributed to FMR, but per se may not cause significant regurgitation [6-8,11,17]. In our study, indices of global LV dilation and dysfunction such as LV size and volume, sphericity index and C-septal distance have been greater and ejection fraction has been lower in those with severe regurgitation; however these parameters were not among the primary determinants of FMR severity. Other studies also suggested that the degree of LV dysfunction and enlargement are not primary predictors of FMR and global sphericity had shown poor correlation with regurgitation [2,6,8,17]. Therefore, local LV remodeling and mitral valve deformation, although related to global LV changes, are the strongest independent predictors of functional MR, and seems to be the major targets for proper operative options.

### Study Limitation

We didnot have gold standard to quantify MR severity. The limitations of PISA for quantitative MR assessment were previously reported, but were minimized in this study because we used both PISA and vena contracta methods for assessing MR severity.

## Abbreviations list

FMR: Functional mitral regurgitation; LV: Left ventricular; DCM: Dilated cardiomyopathy; ICM: Ischemic cardiomyopathy; IPMD: Interpapillary muscle distance; TnD: Tenting distance; TnV: Tenting volume; 2D: 2-Dimensional; PISA: Proximal isovelocity surface area; ERO: Effective regurgitant orifice; VC: vena contracta.
